# Key Immunological Functions Involved in the Progression of Epithelial Ovarian Serous Carcinoma Discovered by the Gene Ontology-Based Immunofunctionome Analysis

**DOI:** 10.3390/ijms19113311

**Published:** 2018-10-24

**Authors:** Cheng-Chang Chang, Kuo-Min Su, Kai-Hsi Lu, Chi-Kang Lin, Peng-Hui Wang, Hsin-Yang Li, Mong-Lien Wang, Cheng-Kuo Lin, Mu-Hsien Yu, Chia-Ming Chang

**Affiliations:** 1 Department of Obstetrics and Gynecology, Tri-service General Hospital, National Defense Medical Center, Taipei 114, Taiwan; obsgynchang@gmail.com (C.-C.C.); aeolusfield@hotmail.com (K.-M.S.); kung568@gmail.com (C.-K.L.); hsienhui@ms15.hinet.net (M.-H.Y.); 2 Department of Medical Research and Education, Cheng-Hsin Hospital, Taipei 112, Taiwan; lionel.lu@gmail.com (K.-H.L.); monglien@gmail.com (M.-L.W.); 3 School of Medicine, National Yang-Ming University, Taipei 112, Taiwan; phwang@vghtpe.gov.tw (P.-H.W.); doc3643h@yahoo.com.tw (H.-Y.L.); 4 Department of Obstetrics and Gynecology, Taipei Veterans General Hospital, Taipei 112, Taiwan; 5 Department of Medical Research, China Medical University Hospital, Taichung 404, Taiwan; 6 Department of Medical Research, Taipei Veterans General Hospital, Taipei 112, Taiwan; 7 Department of Obstetrics and Gynecology, Taoyuan Armed Forces General Hospital, Taoyuan 325, Taiwan; medication0206@gmail.com

**Keywords:** ovarian carcinoma, integrative analysis, gene expression microarray, gene set, machine learning, immunological function

## Abstract

Serous carcinoma (SC) is the most common and lethal subtype of epithelial ovarian carcinoma; immunotherapy is a potential treatment for SC, however, the global immunological functions of SC as well as their change during the progression of SC have not been investigated in detail till now. We conducted a genome-wide integrative analysis to investigate the immunofunctionomes of SC at four tumor stages by quantifying the immunological functions defined by the Gene Ontology gene sets. DNA microarray gene expression profiles of 1100 SCs and 136 normal ovarian tissue controls were downloaded from the Gene Expression Omnibus database and converted to the functionome. Then the immunofunctionomes were reconstructed by extracting the offspring from the functionome for the four SC staging groups. The key immunological functions extracted from immunofunctionomes with a series of filters revealed that the immunopathy of SC consisted of a group of deregulated functions with the core members including B cell activation and differentiation, regulation of leukocyte chemotaxis/cellular extravasation, antigen receptor mediated signaling pathway, T helper mediated immunity and macrophage activation; and the auxiliary elements included leukocyte mediated immunity, regulation of inflammatory response, T cell differentiation, mononuclear cell migration, megakaryocyte differentiation, complement activation and cytokine production. These deregulated immunological functions reveal the candidates to target in the immunotherapy.

## 1. Introduction

Ovarian cancer is the fifth most common cancer type worldwide, and the second most frequent malignant tumor in females, which accounts for almost 3% of cancer in females [1] and also ranks fifth cause of cancer-related deaths among women, accounting for more deaths than any other cancer of the female reproductive system. The Federation of Gynecology and Obstetrics (FIGO) system, the most commonly utilized staging system of serous carcinoma (SC), divides SC into four stages based on the progression of SC [2]. FIGO staging has been widely used to evaluate disease survival or treatment response in many previous clinical studies.

There are several genetic and environment factors that contribute to the development of SC, which considered to be a complex disease with complicated carcinogenesis pathway. Recent genome-wide studies have also greatly increased our understanding of the general molecular pathways implicated in SC. However, the immunologic functions of SC at different FIGO stages have not been quantified or measured within tumors and tumor environment. Immune response and inflammation functions of the host defense system aim to protect the body against internal insults. Inflammation is a critical modulator of carcinogenesis, which is associated with secretion of inflammatory cytokines leading to the formation of an inflammatory microenvironment which is considered to be a hallmark of cancers [3]. Ovarian carcinoma may be recognized and attacked by the immune system and the presence of intra-tumoral T cells correlates with improved progression-free survival and overall survival among patients with ovarian carcinoma and is associated with activation of molecular antitumor mechanisms [4].

Immunological functions can be investigated by the differentially expressed genes (DEGs) detected by microarrays. In contrast to DEGs, we established a gene set regularity model, which reconstructed the functionomes, i.e., the gene set regularity (GSR) indices of the global functions, and then investigated the deregulated functions involved in the complex disease. Establishing the functionome can provide us the information about the deregulated functions for complex diseases [5,6,7,8]. In the past, we have carried out several gene set-based analyses by integrating the microarray gene expression profiles downloaded from the publicly available databases and our previous research. In such a way, it was revealed that deregulation of cell cycle was more predominant in SC, while the Erb-B2 receptor tyrosine kinase (ERBB) and phosphoinositide 3-kinase (PI3K)-related pathways played important roles in the carcinogenesis of clear cell carcinoma, endometrioid carcinoma and mucinous carcinoma [6], and that deregulated oxidoreductase activity, metabolism, hormone activity, inflammatory response, innate immune response and cell-cell signaling play the key roles in the malignant transformation of endometriosis-associated ovarian carcinoma (EAOC) [7].

For years, the foundations of cancer treatment are surgery, chemotherapy, and radiation therapy. But over the past several years, immunotherapy approaches that enlist and strengthen the power of a patient’s immune system to attack tumors [9]. Immunotherapy is one of the multiple therapeutic treatments of cancer, which includes treatments that work in different ways: some boost the body’s immune system in a very general way, while others help train the immune system to attack cancer cells specifically. For some types of cancer, immunotherapy works better than for others. It is used by itself for some of these cancers, but for others it seems to work better when used concomitantly with other types of treatment. Many newer types of immune treatments are now being studied, and they will impact how we treat cancer in the future.

The development of effective immunotherapy approaches is conditional on a thorough understanding of the immunological functions in SC. However, we still do not have clear information about the immunological functions and related effective mechanism at different SC stages although the FIGO staging system reveals great consistence with the progression and disease severity of SC. The knowledge of the mechanisms of deterioration of these functions from stage I to IV of SC will facilitate the investigation of SC pathogenesis. This time, we conducted a whole-genome integrative analysis to investigate the global immunological functions of SC at different stages to explore immunological deterioration during SC progression from FIGO stage I to stage IV by quantifying the immunological functions defined by the GO defined gene sets. The result of this analysis may contribute to the improvement and enhancement of immunotherapy for SC.

## 2. Results

### 2.1. DNA Microarray Gene Expression Datasets for SC and Gene Ontology (GO) Gene Set Definition

A total of 1100 SC samples were collected from the Gene Expression Omnibus (GEO) database, including 34, 39, 695 and 131 samples of stages I, II, III and IV, respectively. 136 normal ovarian samples were collected as control group (Table 1). These samples covered 33 datasets from 5 different DNA microarray platforms. The detailed information on the samples is available in Table S1. The 5917 GO gene set definitions for annotating the functionome were downloaded from the Molecular Signatures Database (MSigDB) [10] with the version of “c5.all.v6.2.symbols.gmt”.

### 2.2. Reconstruction and Comparison of Functionomes Between the SC Groups and Normal Controls

**Figure 1:**
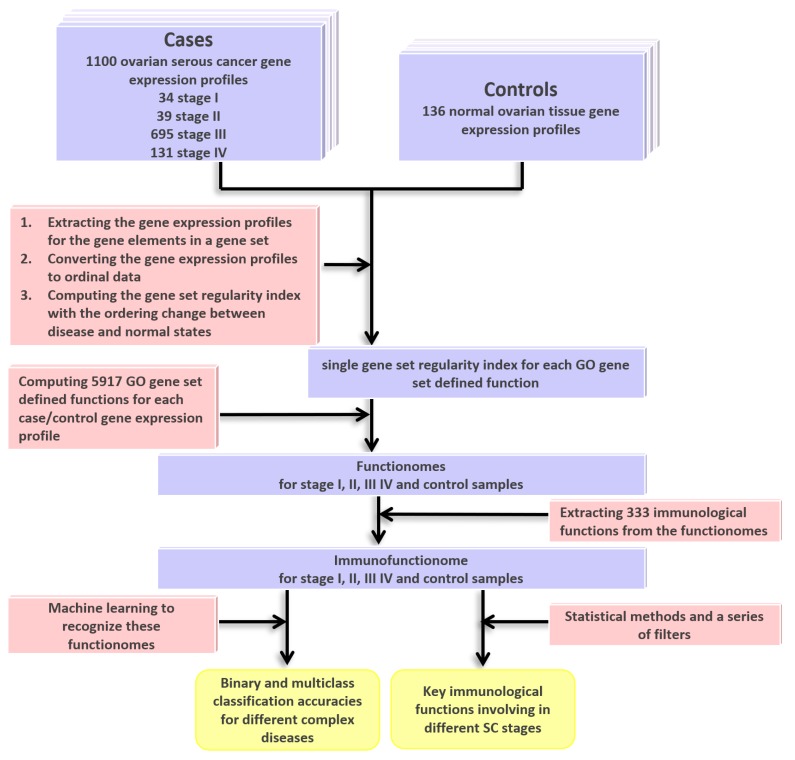
Workflow of this study. The DNA microarray gene expression datasets for the four serous carcinoma (SC) staging groups and normal ovarian controls were downloaded from the publicly available database. The gene set regularity (GSR) index was computed by measuring the changes of the gene expression ordering of the gene elements in the Gene Ontology (GO) gene set. The functionome consisting of 5917 GO gene set defined functions was reconstructed for each sample. Then, the immunofunctionome consisting of 333 immunological functions was reconstructed by extracting the immune-ancestor GO terms from the functionome for the four staging and normal control groups. Machine learning was applied to recognize the patterns of the functionomes and then executed the binary and multiclass classifications. The key immunological functions were extracted by the statistical methodology and a series of filters from the immunofunctionomes.

The DNA microarray gene expression profiles of the four SC stages and normal ovarian samples were converted to the GSR indices by measuring the levels of the ordering changes of the gene elements in the GO gene sets between the SC cases and normal controls. The workflow this study was displayed on Figure 1. The gene expression profiles for the gene elements in a gene set were extracted and converted to ordinal data. Then the GSR index was computed based on the ordering change between disease and normal states. It ranges from 0 to 1, 0 represents the orderings between the case are completely different from the normal state, indicating the most deregulated state of a function. As the first step, functionome consisting of 5917 GO gene set defined functions was reconstructed for each sample, the statistics of the functionomes are listed on Table 1. After corrected by the averages of the control groups, the corrected means of the GSR indices decreased stepwise from 0.6214 in stage I, to 0.6041 in stage II, 0.5715 in stage III and 0.5583 in stage IV, indicating that the steady deterioration of functional regulation as disease progression. The differences in the GSR indices between each SC staging and the normal control groups were statistically significant (*p* < 0.05), indicating that the functions were generally deregulated in the SC groups compared with the normal controls.

It was difficult to precisely extract the complete immune-related GO terms from the GO database directly. To collect them as comprehensive as possible, we utilized the following two ancestor GO terms, including “immune system process” (GO:0002376) and “inflammatory response” (GO:0006954) to reconstruct the immunofunctionome by extracting their 333 offspring from the functionome. 

**Figure 2:**
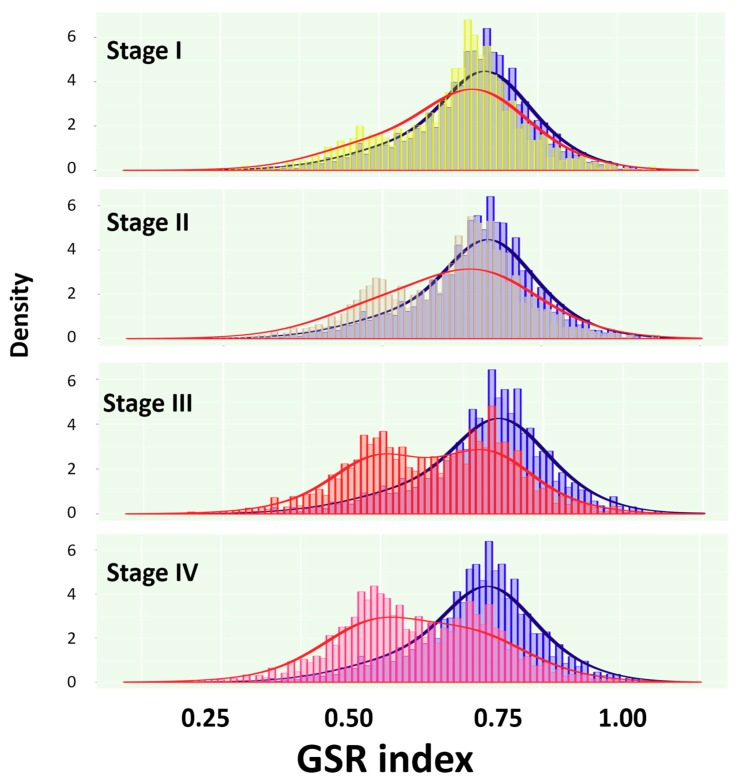
Histograms of the GSR indices for the immunofunctionomes of stage I to IV and control groups. The figures show two different distributions for the immunofunctionomes from the SC stage I to IV and control groups. The normal ovarian tissue group (blue) located on the right side of the histogram was utilized as the controls for the four SC staging groups. A second peak of distribution was observed and increased in density from stage I to IV, indicating a group of deregulated immunological functions growing in number and increasing in severity as SC progression.

When the total GSR indices from the four immunofunctionomes were displayed on the histogram (Figure 2), each SC staging and the normal ovarian tissue groups appeared as two different distributions. As the disease progressed, the two distributions were getting farther apart from each other. Furthermore, a double-peak pattern of the immunofunctionome could be observed since stage III, indicating a group of deregulated immunological functions growing in number and increasing in severity as the disease progressed. 

### 2.3. Comparison of the Immunofunctionomes among the Four SC Staging Groups

**Figure 3:**
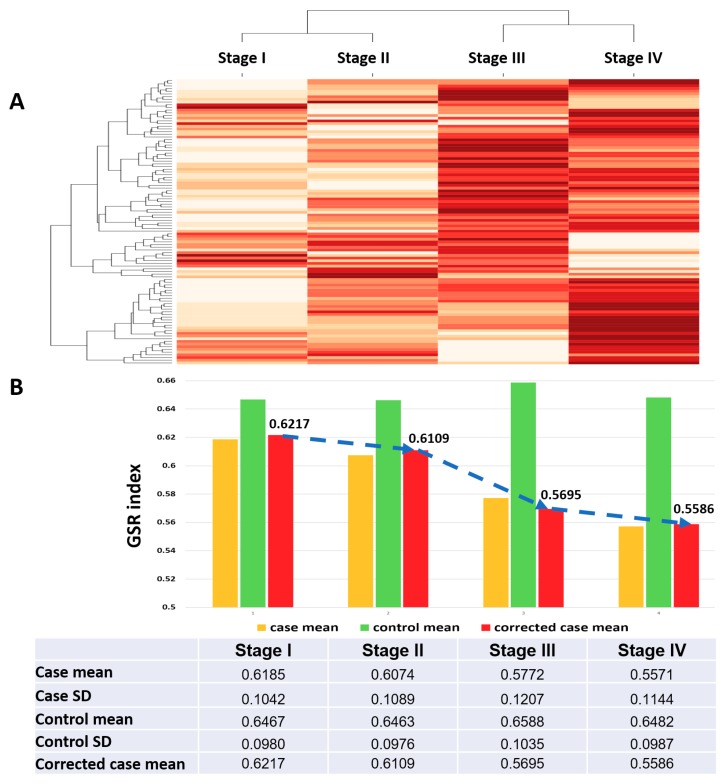
Dendrogram, heatmaps and the means of GSR indices for the stage I–IV immunofunctionome. (**A**) The dendrogram (top of the heatmap) shows the relationship among the four immunofunctionomes, which are correctly classified by unsupervised classification. The heatmap showed the deterioration of function regulation from stage I to IV. (**B**) The values labeling the average of corrected GSR indices for each staging group showed stepwise deterioration from stage I to IV. The mean and SD for the SC samples and controls were listed in the bottom table.

The immunofunctionomes from the four SC staging groups showed distinct patterns and could be accurately recognized and classified with unsupervised classification by hierarchical clustering as shown in Figure 3. Based on their functional regularity patterns, the dendrogram showed the correct order of functionome from stage I to IV (Figure 3A). Progressive deterioration of the immunological function regulation from stage I to IV can be visualized by the patterns of the heatmap (Figure 3A). After quantifying the regulation of immunological functions by measuring the average of the total GSR indices in each immunofunctionome and then corrected by the means of the control groups, the levels of the corrected GSR indices for stage I, II, III and IV were 0.6217, 0.6109, 0.5695 and 0.5586, respectively, showing the regularity of global immunological functions deteriorating stepwise during disease progression.

### 2.4. The Global Function Regulation from Stage I to IV Shows Distinct Pattern That Can Be Correctly Classified and Predicted by Machine Learning

We utilized support vector machine (SVM), a supervised machine learning algorithm to recognize, classify and predict the distinct patterns among the four staging groups. The performance was tested by k-fold cross-validation with the sensitivities, specificities and accuracies of the binary and multiclass classifications. For example, when k = 3, meaning that the data are divided into three parts, two parts are utilized as a training set, and the remaining part as a test set to predict the results of classification. The performances listed in Table 2 were the result of the averages of 10 successive classifications and predictions. The results showed the accuracies were 100% in stage I, III and IV with 5-fold cross-validation. The accuracy of the multiclass classification among the stage I–IV groups was 93.38%. This decreased accuracy probably arose from the similarities in the functional regularity among the four staging groups. These results revealed that the functions, as quantified by the GSR indices converted from the microarray gene expression profiles, could provide sufficient information for machine learning to recognize and perform correct classification. These results also indicated that GSR indices could be utilized for molecular classification among gene expression profiles from different FIGO stages of SC.

### 2.5. The Most Significantly Deregulated Immunological Fucntions for the Four SC Staging Groups

The statistically significant immunological functions in the immunofunctionomes were ranked by their *p* values to show the deregulated immunological functions from stage I to IV. Table 3 shows the top 20 deregulated immunological functions from stage I to IV. The full list is available in Table S2. The most deregulated immunological functions for each stage were “positive regulation of B cell mediated immunity”, “regulation of B cell mediated immunity”, “negative regulation of CD4 positive αβ T cell activation”, and “regulation of B cell mediated immunity”. In general, the most deregulated functions were related to T and B lymphocytes. 

### 2.6. The Commonly Deregulated Immunological Functions among the Four Staging Groups

**Figure 4:**
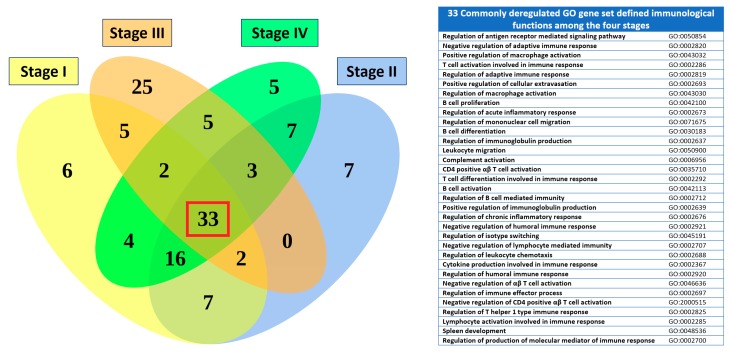
Venn diagram of the commonly deregulated immunological functions for the four SC staging groups. The results of the set analysis of the stage I–IV groups with the top 75 significantly deregulated immunological functions are displayed on the Venn diagram to show the gene set numbers of all possible logical relations among the stage I to IV groups. There were 33 common GO terms among the four staging groups, listed on the right-side table.

Because many deregulated immunological functions appeared repeatedly among different stages in Table 3, we carried out the set analysis to find out the commonly deregulated immunological functions among the four SC staging groups. The commonly deregulated GO gene set defined functions were selected by intersecting the top 75 statistically significant immunological functions among the four staging groups. The results showed the commonly deregulated immunological functions as shown in Figure 4. There were 33 common GO terms among the four staging groups, generally associated with T cells, B cells mediated immunity, antigen receptor mediated signaling pathway, leukocyte chemotaxis, cellular extravasation, cytokine production and macrophage activation.

### 2.7. The Progressively Deregulated Immunological Functions in the Pathogenesis of SC from Stage I to IV

**Figure 5:**
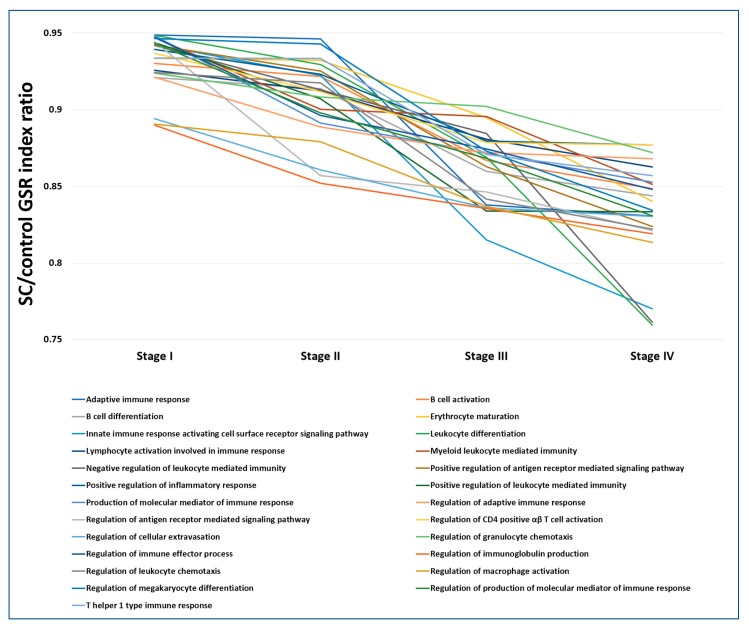
The progressively deregulated GO gene set defined immunological functions from SC stage I to IV. The GO gene set defined immunological functions that were statistically significant and decreased in the GSR index levels from SC stage I to IV were selected. The progressively deregulated immunological functions among the four staging groups were compared by the SC/control GSR index ratio, a ratio of GO function normalized by the corresponding normal control group. A total of 25 GO terms met the criteria as the bottom list shown.

In addition to filtering the commonly deregulated functions among the four stages, we also extracted the crucial immunological functions involved in the disease progression by selecting functions whose GSR index level decreased progressively from stage I to IV. The progressively deregulated immunological functions among the four staging groups were compared by the SC/control GSR index ratio, a ratio of GO function normalized by the corresponding normal control group. As shown in Figure 5, there were 25 progressively deregulated immunological functions that met this selection criteria. These GO genes set defined functions were associated with leukocyte mediated immunity, T and B cell mediated immunity, antigen receptor mediated signaling pathway, leukocyte chemotaxis, cellular extravasation, inflammatory response and macrophage activation.

### 2.8. The Core and Auxiliary Elements of Deregulated Immunological Functions Involved in the Progression of SC

**Figure 6:**
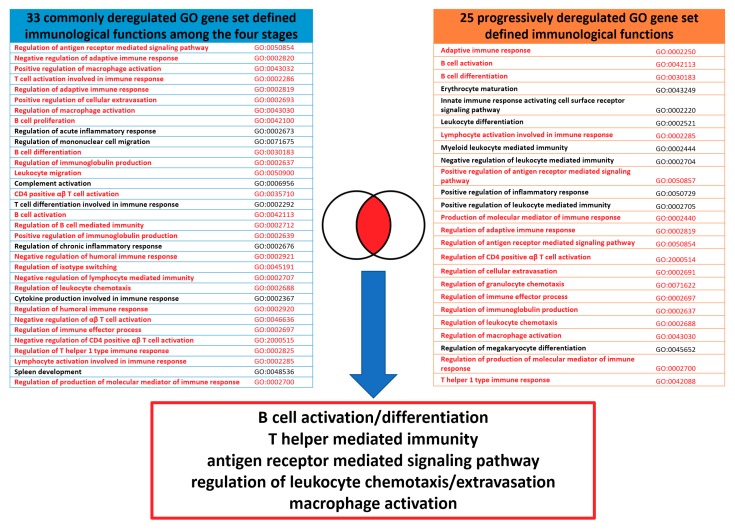
The core elements of the immunofunctionome involved in the progression of SC from stage I to IV. The five core elements of the deregulated immunological functions were extracted based on the GO semantic similarities between the deregulated GO gene set defined functions among the four staging groups, and the progressively deregulated immunological functions from stage I to IV as the bottom box listed. The similar or matched GO terms between the two groups were marked in red.

Finally, the core elements of the deregulated immunological functions were extracted by merging based on the GO semantic similarities between the deregulated GO gene set defined functions among the four staging groups, and the progressively deregulated immunological functions from stage I to IV. The non-specific, upper-level GO terms were not included. As the Figure 6 shown, the core elements could be summarized as the following five immunological functions: (1) B cell activation and differentiation, including “B cell activation” (GO:0042113), “B cell differentiation” (GO:0030183), “regulation of immunoglobulin production” (GO:0002637) and “positive regulation of immunoglobulin production” (GO:0002639); (2) regulation of leukocyte chemotaxis/cellular extravasation, including “regulation of leukocyte chemotaxis” (GO:0002688), “regulation of granulocyte chemotaxis” (GO:0071622), “leukocyte migration” (GO:0050900), “regulation of cellular extravasation” (GO:0002691) and “positive regulation of cellular extravasation” (GO:0002693); (3) T helper mediated immunity, including “regulation of CD4 positive αβ T cell activation” (GO:2000514), “T helper 1 type immune response” (GO:0042088), “CD4 positive αβ T cell activation” (GO:0035710), “negative regulation of CD4 positive αβ T cell activation” (GO:2000515), “negative regulation of αβ T cell activation” (GO:0046636), and “regulation of T helper 1 type immune response” (GO:0002825); (4) antigen receptor mediated signaling pathway, including “positive regulation of antigen receptor mediated signaling pathway” (GO:0050857) and “regulation of antigen receptor mediated signaling pathway” (GO:0050854); (5) macrophage activation, including “regulation of macrophage activation” (GO:0043030) and “positive regulation of macrophage activation” (GO:0043032).

The auxiliary elements of the deregulated immunological functions were extracted from the symmetric difference between the deregulated GO gene set defined functions among the four staging groups, and the progressively deregulated immunological functions from stage I to IV. The auxiliary elements could be summarized as the following five immunological functions: (1) leukocyte mediated immunity, including “leukocyte differentiation” (GO:0002521), “myeloid leukocyte mediated immunity” (GO:0002444), “negative regulation of leukocyte mediated immunity” (GO:0002704) and “positive regulation of leukocyte mediated immunity” (GO:0002705); (2) regulation of inflammatory response, including “regulation of acute inflammatory response” (GO:0002673), “regulation of chronic inflammatory response” (GO:0002676) and “positive regulation of inflammatory response” (GO:0050729); (3) T cell differentiation, including “T cell differentiation involved in immune response” (GO:0002292) and “T cell activation involved in immune response” (GO:0002286); (4) mononuclear cell migration (“regulation of mononuclear cell migration” (GO:007167)); (5) megakaryocyte differentiation (“regulation of megakaryocyte differentiation” (GO:0045652)); (6) cytokine production (“cytokine production involved in immune response” (GO:0002367)); (7) complement activation (“complement activation” (GO:0006956)).

### 2.9. The Differentially Expressed Genes in the Core Elements of Deregulated Immunological Functions Involved in the Progression of SC

**Figure 7:**
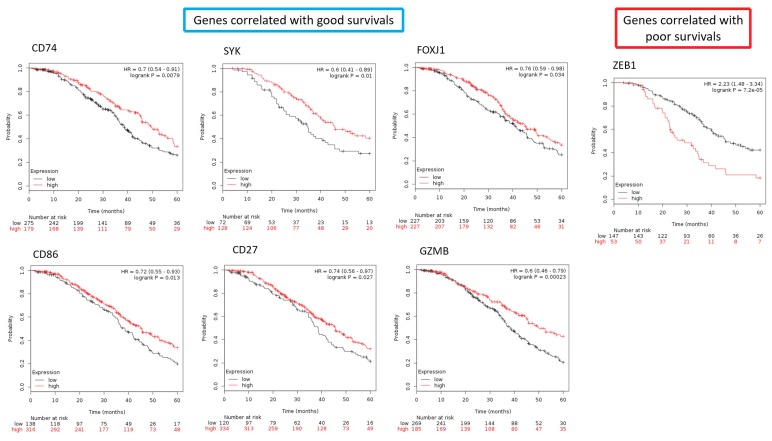
The correlation between the SC survivals and the immunological genes involved in macrophage activation, T-cell differentiation and lymphocyte mediated immunity. High expression levels of six immunological genes (*CD74*, *SYK*, *FOXJ1*, *CD86*, *CD27*, *GZMB*) tend to correlate with good patient survival; in contrast, high expression levels of *ZEB1* is correlated with poor survival with statistical significance.

To further illustrate the role of key immunological genes involved in in SC survival, we used Kaplan–Meier plotter (http://www.kmplot.com/ovar) to explore the correlation between SC patient survival and the expression levels of the DEGs in the core elements of deregulated immunological functions involved in the progression of SC. To obtain the list containing all possible immunological genes, we utilized the gene list provided by the innateDB [11], a database collecting a relatively comprehensive immune-related gene list. After filtering the DEGs in the core elements of deregulated immunological functions involved in the progression of SC with this gene list, we selected 26 most significantly, immune-related DEGs for the four staging groups, including 8 macrophage activation-related genes (*CD74*, *WNT5A*, *NR1H3*, *STAP1*, *RORA*, *ZC3H12A*, *PLA2G10*, *IL33*), 9 genes involved in T-cell differentiation (*SYK*, *MYB*, *FOXJ1*, *ZEB1*, *CD74*, *LGALS9*, *ADAM8*, *GLI2*, *CD86*) and 9 genes involved in lymphocyte-mediated immunity (*PRKCD*, *PTPN6*, *MSH2*, *EXO1*, *CD74*, *SLC11A1*, *CD27*, *GATA3*, *GZMB*). Then we correlated the gene expression with the SC patient survival outcome with the database created by Gyorffy et al. [12]. It is an online tool to assess the prognostic value of the expression levels of all microarray-quantified genes in ovarian cancer patients with the gene expression profiles and survival information of 1287 ovarian cancer patients downloaded from the NCBI, included GSE3149, GSE9891, GSE14767, GSE15622, GSE18520, GSE19829, GSE23554, GSE26193, GSE26712, GSE27651, GSE30161, GSE51373, GSE63885, GSE65986, and TCGA. We analyzed expression for overall survival in serous FIGO I/II/III/IV EOC patients with chemotherapy of platin + taxane. After quality control and normalization, only probes present on all three Affymetrix platforms (Affymetrix HG-U133A, HG-U133A 2.0, and HG-U133 Plus 2.0 microarrays) were retained (*n* = 22,277); all possible cutoff values between the lower and upper quartiles are computed, and the best performing threshold is used as a cutoff. We correlated the gene expression levels of 26 immunological genes with SC patient survival outcome. We found that high expression levels of 6 immunological genes (*CD74*, *SYK*, *FOXJ1*, *CD86*, *CD27*, *GZMB*) tend to correlate with good patient survival with statistical significance (Figure 7). There was one immunological gene, *ZEB1*, whose high expression level correlated with poor survival with statistically significance (Figure 7). The hazard ratios of *CD74*, *SYK*, *FOXJ1*, *CD86*, *CD27*, *GZMB* were 0.7(0.54–0.91, *p* = 0.0079), 0.6(0.41–0.89, *p* = 0.01), 0.76(0.59–0.98, *p* = 0.034), 0.72(0.55–0.93, *p* = 0.013), 0.74(0.56–0.97, *p* = 0.027), 0.6(0.46–0.79, *p* = 0.00023), respectively; the hazard ratios of *ZEB1* is 2.23(1.48–3.34, *p* = 0.00007) (Figure 7). These results suggested key roles of the macrophage activation, T-cell differentiation and lymphocyte mediated immunity in promoting SC progression, as well as their prognostic value in SC. The full DEGs list was available in Table S3.

## 3. Discussion

After converting to the GSR indices, our results showed clear stepwise deterioration of the global immunological functions from stage I to IV. The histogram revealed the presence of a group of deregulated immunological functions that increased in severity and number from stage I to IV, which were investigated in the subsequent studies. We demonstrated the patterns of functionomes were distinct and could be precisely recognized and classified by unsupervised classification with hierarchical clustering and by supervised classification using SVM. These results revealed that the informativeness of the GSR indices was sufficient to make a clear distinction among the patterns of the four stages. In this study, the most deregulated immunological functions in SC ordered by statistical significance were associated with regulation of T and B lymphocyte mediated immunity.

To explore the deregulated immunological functions involved in the progression of SC, we extracted the commonly deregulated immunological functions among the four staging groups, as well as the progressively deregulated immunological functions as SC progression from stage I to IV. The core elements involved in the SC progression were further extracted by detecting the common part between these two deregulated functions based on their GO semantic similarities, including B cell activation and differentiation, regulation of leukocyte chemotaxis/cellular extravasation, antigen receptor mediated signaling pathway, T helper mediated immunity and macrophage activation; and the auxiliary elements included leukocyte mediated immunity, regulation of inflammatory response, T cell differentiation, mononuclear cell migration, megakaryocyte differentiation, complement activation and cytokine production. These deregulated immunological functions reveal the immunopathy the candidates to target in the immunotherapy for SC.

T cell mediated immunity was the most significantly deregulated and the core element of SC progression detected in this study. It is well-known that T cells played a central role in immune-editing within epithelial ovarian cancer tumors and tumor environments. With or without this specific population of T cells is associated with significant differences in prognosis of ovarian cancers. As previously stated, analysis of the tumor microenvironment in patients with a variety of solid tumors showed that a substantial subset of tumors with evidence of a T cell–infiltrated phenotype [13]. Tumor-associated antigens (TAAs) are one of the initial triggers of the immune response. They are crucial because they can activate the T cell response via major histocompatibility complex (MHC), which is an essential branch of defense mechanism against tumorigenesis [14]. Studies in paraffin-embedded tissues have substantiated this concept and have shown that the presence of tumor infiltrating lymphocytes (TIL) such as CD3+ cells and an elevated number of cytotoxic CD8 lymphocytes were connected with prolongation of survival [13]. For example, patients with EOC presenting higher CD3 cell numbers had a prolonged overall survival of 60 months over 29 months for patients that had lower CD3 cell numbers [15].

Macrophages were detected as the core element involved in SC progression in this study. The existence of macrophages in tumors has been associated with tumor growth and metastasis in rodents at first [16]. Macrophages and other similar sorts of myeloid cells are found in the microenvironment of solid tumor universally and can contribute to immune evasion eventually. Moreover, increased number of tumor-associated macrophages (TAMs) may be involved in enhanced tumor neovascularization, associating with poor patient prognosis and tumor resistance to therapies. Co-culturing of ovarian cancer cell lines with TAMs improves endothelial cell migration and tube formation, as well as the accumulation of several pro-angiogenic cytokines, including growth factors and inflammatory cytokines or mediators. No matter during tumor growth or in response to cytotoxic therapy, TAMs can enhance tumor revascularization (e.g., radiotherapy), thereby causing cancer relapse [17].

T helper 1 type and cytokine production involved in immune response were the core and auxiliary elements of SC progression, respectively. These two functions are close related. Many cytokines have been either associated with a direct effect on tumor cells via surface receptors, such as Toll-like receptors, or they have accessorial roles in supporting the immune response against tumors. The antitumor response of host results from the balance between the T helper 1 (Th1) response, which makes the immune response more powerful and the T helper 2 (Th2) responses characterizing oncogenesis and disease progression with a shift in favor of the latter. Both Th1 and Th2 immune responses have been associated with the production of cytokines, such as interleukin 12 (IL-12), interleukin 4 (IL-4), interferon gamma (IFN-γ), tumor necrosis factor (TNF-α) (Th1 response), and interleukin 10 (IL-10) (Th2 response). Cancer cells, present in tumor tissue, peripheral tumor microenvironments and even in ascites, can also produce these cytokines which have been proved to be associated with prognosis in ovarian cancer [13,18].

Three key deregulated functions involved in the SC progression, including antigen receptor mediated signaling pathway, T cell differentiation and B cell mediated immunity, were related to immunosurveillance. Immunosurveillance has been recognized as an essential component of host anticancer reaction for a long time. Agents which can aggrandize immune response, as well as antibodies against specific tumor-associated antigens, have been approved for the treatment of different types of tumors, including ovarian cancers [19]. The immune system responds to the presence of cancer antigens. Most tumor cells express antigens that can induce recognition by host CD8+ T cells [20]. Cancers that are detected clinically must have evaded antitumor immune responses to grow progressively. A recent critical advance in immunology has been the elucidation of antigen-specific cell recognition and destruction of target cells. The innate and adaptive immune responses are equipollent influentially in the battle field against ovarian cancer. Equilibrium and elimination are reached via lymphocytes, mostly the T cell subpopulation [13]. Recent studies also support these concepts mainly showing that the presence of tumor infiltrating lymphocytes may be associated with better prognosis and clinical outcome in patients with cancer including ovarian carcinoma [13]. Although the progress of antitumor immune response has been established, there are also evidence and review that tumors can escape destruction by suppressing the immune system both within the cancer microenvironment and on a systemic level [21]. It is well proposed that the presence or absence of specific populations of T cells, a key role in immune-editing within epithelial ovarian cancer (EOC), is associated with essential differences in prognosis [22]. In recent years, in addition to the well-established role of regulatory T cells in forming anti-tumor immunity, a new wave of research has described an emerging role of B cells with immunosuppressive and/or regulatory functions in modulating anti-tumor immune responses and in carcinogenesis. B-cell subsets with specific phenotypes and functions may also possess multiple roles in relation to anti-tumor responses [23]. As a result, regulation of lymphocyte-mediated immunity and adaptive immune response takes place in critical immunological function in ovarian cancer development.

The immunological imbalance between activation and suppression may result in oncogenesis and cancer progression. Natural killer (NK) cells exist in the blood as pre-activated cytolytic lymphocytes and are identified as the most efficient antitumor effectors. The macrophages, one of the key elements in the immunopathy of SC, is known as the essential to increase the anti-tumor activity of NK cells through their crosstalk [24]. 

This research focuses on “function” instead of “gene”. However, because each GO gene set is defined by a group of genes, we also checked the important immune-related genes in the gene set of the key immunological functions involved in the progression of SC. The *ZEB1* gene was predicted to be associated with poor prognosis by the KM plotter in this study. A clinical study has demonstrated the high expression of *ZEB1* was associated with recurrence and progression-free survival and concluded the positive *ZEB1* expression may be an indicator of unfavorable progression-free survival in patients with EOCs [25]. However, the role of *ZEB1* in the immune system and SC progression needs to be clarified in the future.

Exploiting the immune system has been proved to be a practical therapeutic approach in treating a variety of malignancies [26]. Immune cells infiltrating the tumor tissue are associated positively or negatively with antitumor activity. Investigating the relationship of a network between tumor microenvironment, immune cells interact with tumor cells, and each other will considerably promote the advance of more useful immunotherapies for ovarian cancer [27].

The pathogenesis of a complex disease, such as SC, is usually involved in multiple genes and their interactions. Traditionally, the workflow of analyzing microarray gene expression data is focusing on detection of DEGs and then mapping them to the GO terms or pathways for the enrichment analysis to identify the aberrant functions. This approach focuses on the statistically significant genes or functions, but those genes that do not reach significance criteria are omitted. In addition, the gene-gene interactions are usually not included in such calculation. Based on these limitations, we utilized the polygenetic, GO gene set-based model to investigate the immunopathy of SC. The aberrant immunological functions were investigated by analyzing the immunofunctionomes consisted of 333 generalized immunological functions reconstructed by extracting the offspring from the upmost immune-related ancestor GO terms from the functionomes. Computing the GSR indices will take the interactions of the gene elements in a gene set into account. In addition, the reduction of data dimension from tens of thousands to 5917 will reduce data noise. This workflow is able to provide a more comprehensive and intuitive way to investigate the immunopathy of SC. 

This model has limitations. The first limitation is that the GO gene set databases do not collect all human functions yet. The second limitation is the detectability of the GSR model. Because this model converts gene expression levels to ordinal data, the GSR index will remain unchanged and aberrations will be missed if the expression levels do not reach the detection levels. The third limitation is the false positivity arising from the duplicated elements existing in different gene sets. The fourth limitation comes from the heterogenicity of cellular composition in tumor and control samples. The datasets utilized in study are composed of the gene expression profiles from the mixture of immune and tumor cells. So, the differences of GSR indices may arise from the gene expressions of differing sampled cellular compositions and may not exactly reflect a deregulated process. The fifth limitation is the fluctuation of control GSR indices among the four staging groups. The functionomes were reconstructed by integrating numerous datasets in the “SC-control pair” style using the common genes between the two groups. Because the DNA microarray platforms varies among these SC datasets, the gene lists may differ and lead to fluctuation of the control GSR indices even though the same group of normal ovarian samples was utilized for the four staging groups. To fix this bias, we normalized the SC GSR indices by the control data before comparison among the four staging groups.

## 4. Materials and Methods

### 4.1. Computing the GSR Indices and Reconstruction of Functionome and Immunofunctionome

The GSR index is computed from the gene expression profiles by modifying the differential rank conservation (DIRAC) [28] algorithm, which measures the changes of the ordering among the gene elements in a gene set between the gene expression profiles of SC and the most common gene expression ordering in the normal control population. The detail of the GSR model and the computing procedures are described in our previous study [5]. Microarray gene expression profiles for SC and normal ovarian samples were downloaded from the GEO database. The corresponding gene expression levels were extracted according to the gene elements in the GO gene set and converted to the ordinal data based on their expression levels. The GSR index is the ratio of gene expression ordering in a gene set between the case and the most common gene expression ordering among the normal ovarian samples. Measurement of GSR indices is executed in the R environment. A functionome is defined as the complete set of biological functions. At present, the definition for comprehensive biological functions is not yet available, so we annotated the human functionome by the 5917 GO gene set defined functions. The functionome in this study is defined as the assembly of 5917 GSR indices for each sample. Then the immunofunctionome was reconstructed by extracting the offspring from the immune-related ancestor GO terms “immune system process” (GO:0002376) and “inflammatory response” (GO:0006954) from the functionome.

### 4.2. Microarray Datasets Collection

The selection criteria for the microarray gene expression datasets from the GEO database is listed as follows: (1) the SC samples and normal control samples should originate from the ovarian tissue; (2) the datasets should provide information about the diagnosis and the stage of SC; and (3) any gene expression profile in a dataset was discarded if it contained missing data.

### 4.3. Statistical Analysis

The differences between the SC staging groups and the controls were tested by the Mann-Whitney *U* test, then corrected by multiple hypotheses using the false discovery rate (Benjamini-Hochberg procedure). The p value was set at <0.05.

### 4.4. Classification and Prediction by Machine Learning

The function “ksvm” provided by the “kernlab” (version 0.9–27, The Comprehensive R Archive Network), an R package for kernel-based machine-learning methods was used to classify and predict the patterns of the GSR indices. The accuracies of the classification and predictions by SVM were measured by k-fold cross-validation The performance of binary classification was assessed by results of 10 repeated predictions. AUC was computed using the R package “pROC” [29]. The performance of multiclass classification was assessed by the 10 repeated prediction accuracies for the four SC staging groups. 

### 4.5. Set Analysis

All possible logical relations among the deregulated gene sets of the four SC staging groups were displayed in the Venn diagram using the R package “VennDiagram” (version 1.6.16, The Comprehensive R Archive Network).

## 5. Conclusions

**Figure 8:**
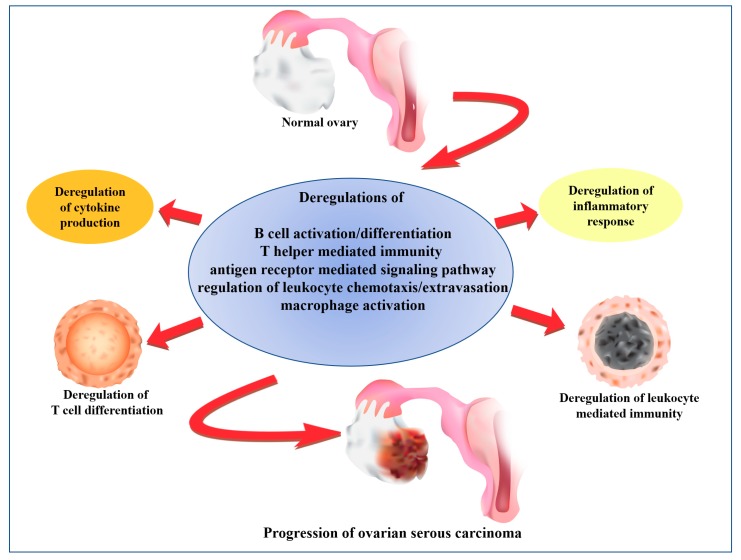
The immunopathy of SC progression.

Immunotherapy has shown to be a promising therapy for many cancers. For the development of effective immunotherapy, a thorough understanding of the immunological functions of a cancer is necessary. Because the regulatory state of the immunological functions as the progression of SC is limited, we conducted a genome-wide integrative analysis to investigate the global immunological functions among the four stages of SC by reconstruction of the immunofunctionomes. The results revealed the immunological function regularity showed a stepwise deterioration, consistent with the severity of SC associated with the four FIGO stages. To summarize the complicated immunopathy of SC, we utilized a series of filters to extract the key members of the immunopathy from the immunofunctionomes. The results revealed the immunopathy of SC consisted of a group of deregulated functions with the core members including B cell activation and differentiation, regulation of leukocyte chemotaxis/cellular extravasation, antigen receptor mediated signaling pathway, T helper mediated immunity and macrophage activation; and the auxiliary elements included leukocyte mediated immunity, regulation of inflammatory response, T cell differentiation, mononuclear cell migration, megakaryocyte differentiation, complement activation and cytokine production. Based on our data-driven analysis, we proposed a working model of the association between immunological deterioration in the progression of SC (Figure 8). These deregulated immunological functions provide us potential targets in the immunotherapy for SC. 

## Figures and Tables

**Table 1:** Sample number and the statistics of the functionomes for the four SC staging groups.

Stage	Case	Control	Total	Case Mean (SD)	Control Mean (SD)	Corrected Case Mean	*p* Value *
I	34	136	170	0.6195 (0.1035)	0.6461 (0.1018)	0.6214	<0.05
II	39	136	175	0.6021 (0.1109)	0.6459 (0.1017)	0.6041	<0.05
III	695	136	831	0.5748 (0.1205)	0.6518 (0.1083)	0.5715	<0.05
IV	131	136	267	0.5588 (0.1154)	0.6486 (0.1031)	0.5583	<0.05

SD, standard deviation.

**Table 2:** Performance of the binary and multiclass classifications and predictions by machine learning.

Binary Classification	K =	Sensitivity (Mean)	Sensitivity (SD)	Specificity (Mean)	Specificity (SD)	Accuracy (Mean)	Accuracy (SD)	AUC
stage I	5	1.0000	0.0000	1.0000	0.0000	1.0000	0.0000	1.0000
	3	0.9851	0.0313	1.0000	0.0000	0.9964	0.0073	0.9911
	2	0.9933	0.0210	1.0000	0.0000	0.9988	0.0037	0.9969
stage II	5	0.9334	0.0839	1.0000	0.0000	0.9857	0.0150	0.9702
	3	0.9607	0.0422	1.0000	0.0000	0.9913	0.0090	0.9803
	2	0.9711	0.0249	1.0000	0.0000	0.9931	0.0059	0.9852
stage III	5	1.0000	0.0000	1.0000	0.0000	1.0000	0.0000	1.0000
	3	1.0000	0.0000	0.9977	0.0071	0.9996	0.0011	0.9988
	2	0.9997	0.0009	0.9917	0.0148	0.9983	0.0025	0.9955
stage IV	5	1.0000	0.0000	1.0000	0.0000	1.0000	0.0000	1.0000
	3	0.9958	0.0087	0.9976	0.0073	0.9966	0.0054	0.9966
	2	0.9938	0.0103	0.9969	0.0097	0.9954	0.0063	0.9954
Multiclass classification	5	0.9244	0.0227	1.0000	0.0000	0.9338	0.0195	0.9881

AUC, area under the curve; SD, standard deviation.

**Table 3:** The 20 most deregulated GO gene set defined functions for the four SC staging groups ranked by their p values.

	**Stage I**	***p* Value**	**GO Index**
1	Positive regulation of B cell mediated immunity	1.2874 × 10^−14^	GO:0002714
2	T cell differentiation involved in immune response	1.7204 × 10^−14^	GO:0002292
3	Regulation of B cell mediated immunity	3.5164 × 10^−14^	GO:0002712
4	Cytokine production involved in immune response	3.5232 × 10^−14^	GO:0002367
5	Regulation of isotype switching	2.4920 × 10^−12^	GO:0045191
6	Negative regulation of CD4 positive αβ T cell activation	4.4483 × 10^−12^	GO:2000515
7	Negative regulation of αβ T cell differentiation	5.4946 × 10^−11^	GO:0046639
8	Regulation of lymphocyte chemotaxis	9.0873 × 10^−11^	GO:1901623
9	Positive regulation of immunoglobulin production	3.1226 × 10^−10^	GO:0002639
10	Negative regulation of αβ T cell activation	8.6150 × 10^−10^	GO:0046636
11	Positive regulation of activated T cell proliferation	1.2196 × 10^−09^	GO:0042104
12	Negative regulation of adaptive immune response	2.2585 × 10^−09^	GO:0002820
13	Positive regulation of adaptive immune response	3.5157 × 10^−09^	GO:0002821
14	Regulation of immunoglobulin production	3.7150 × 10^−09^	GO:0002637
15	Regulation of adaptive immune response	4.6564 × 10^−09^	GO:0002819
16	T cell activation involved in immune response	6.1231 × 10^−09^	GO:0002286
17	Regulation of macrophage activation	1.0046 × 10^−08^	GO:0043030
18	Regulation of lymphocyte mediated immunity	1.7703 × 10^−08^	GO:0002706
19	Regulation of activated T cell proliferation	1.8826 × 10^−08^	GO:0046006
20	Positive regulation of lymphocyte mediated immunity	6.7086 × 10^−08^	GO:0002708
	**Stage II**		
1	Regulation of B cell mediated immunity	7.3615 × 10^−16^	GO:0002712
2	Positive regulation of B cell mediated immunity	8.6942 × 10^−16^	GO:0002714
3	Regulation of isotype switching	1.6517 × 10^−14^	GO:0045191
4	Cytokine production involved in immune response	2.7748 × 10^−13^	GO:0002367
5	T cell differentiation involved in immune response	1.8020 × 10^−11^	GO:0002292
6	Negative regulation of CD4 positive αβ T cell activation	6.5398 × 10^−11^	GO:2000515
7	Positive regulation of immunoglobulin production	6.5398 × 10^−11^	GO:0002639
8	Regulation of immunoglobulin production	7.4799 × 10^−11^	GO:0002637
9	Regulation of adaptive immune response	1.0861 × 10^−10^	GO:0002819
10	Negative regulation of adaptive immune response	1.3364 × 10^−10^	GO:0002820
11	Positive regulation of adaptive immune response	1.9411 × 10^−10^	GO:0002821
12	Regulation of lymphocyte chemotaxis	1.9411 × 10^−10^	GO:1901623
13	Regulation of lymphocyte mediated immunity	1.2403 × 10^−09^	GO:0002706
14	Negative regulation of αβ T cell activation	7.1847 × 10^−09^	GO:0046636
15	Positive regulation of activated T cell proliferation	8.7581 × 10^−09^	GO:0042104
16	Positive regulation of lymphocyte mediated immunity	8.7581 × 10^−09^	GO:0002708
17	Regulation of humoral immune response	8.7581 × 10^−09^	GO:0002920
18	Positive regulation of macrophage activation	1.0451 × 10^−08^	GO:0043032
19	Negative regulation of humoral immune response	1.8493 × 10^−08^	GO:0002921
20	Regulation of macrophage activation	1.8493 × 10^−08^	GO:0043030
	**Stage III**		
1	Negative regulation of CD4 positive αβ T cell activation	1.3948 × 10^−62^	GO:2000515
2	Negative regulation of αβ T cell activation	3.3724 × 10^−54^	GO:0046636
3	Negative regulation of adaptive immune response	7.1266 × 10^−49^	GO:0002820
4	Erythrocyte homeostasis	1.0745 × 10^−46^	GO:0034101
5	Myeloid cell homeostasis	2.2732 × 10^−45^	GO:0002262
6	T cell differentiation involved in immune response	1.3835 × 10^−43^	GO:0002292
7	MyD88 dependent Toll like receptor signaling pathway	2.4472 × 10^−43^	GO:0002755
8	Regulation of humoral immune response	2.3395 × 10^−41^	GO:0002920
9	B cell proliferation	3.3633 × 10^−41^	GO:0042100
10	Regulation of CD4 positive αβ T cell activation	1.4256 × 10^−40^	GO:2000514
11	T cell activation involved in immune response	7.6933 × 10^−40^	GO:0002286
12	Lymphocyte activation involved in immune response	1.0932 × 10^−39^	GO:0002285
13	Regulation of T helper 1 type immune response	1.2770 × 10^−39^	GO:0002825
14	Natural killer cell activation involved in immune response	3.0394 × 10^−39^	GO:0002323
15	Regulation of αβ T cell proliferation	3.0394 × 10^−39^	GO:0046640
16	Dendritic cell differentiation	4.1689 × 10^−39^	GO:0097028
17	Regulation of lymphocyte chemotaxis	4.1689 × 10^−39^	GO:1901623
18	Erythrocyte development	6.5301 × 10^−39^	GO:0048821
19	Negative regulation of lymphocyte mediated immunity	1.5062 × 10^−38^	GO:0002707
20	Thymic T cell selection	4.8521 × 10^−38^	GO:0045061
	**Stage IV**		
1	Regulation of B cell mediated immunity	4.2582 × 10^−36^	GO:0002712
2	Positive regulation of B cell mediated immunity	1.3571 × 10^−33^	GO:0002714
3	Regulation of isotype switching	1.1468 × 10^−32^	GO:0045191
4	Positive regulation of immunoglobulin production	2.3588 × 10^−29^	GO:0002639
5	Regulation of immunoglobulin production	2.3588 × 10^−29^	GO:0002637
6	Negative regulation of adaptive immune response	5.4043 × 10^−29^	GO:0002820
7	Regulation of adaptive immune response	1.4043 × 10^−28^	GO:0002819
8	T cell differentiation involved in immune response	2.3196 × 10^−28^	GO:0002292
9	Cytokine production involved in immune response	2.4258 × 10^−28^	GO:0002367
10	Regulation of humoral immune response	2.4258 × 10^−28^	GO:0002920
11	Negative regulation of CD4 positive αβ T cell activation	8.1639 × 10^−28^	GO:2000515
12	Regulation of lymphocyte mediated immunity	2.6040 × 10^−27^	GO:0002706
13	Negative regulation of lymphocyte mediated immunity	8.0954 × 10^−26^	GO:0002707
14	Positive regulation of adaptive immune response	8.0954 × 10^−26^	GO:0002821
15	Regulation of acute inflammatory response	8.0954 × 10^−26^	GO:0002673
16	Regulation of T helper 1 type immune response	1.3689 × 10^−25^	GO:0002825
17	Regulation of macrophage activation	2.7349 × 10^−25^	GO:0043030
18	Negative regulation of αβ T cell activation	3.6163 × 10^−25^	GO:0046636
19	Regulation of immune effector process	1.6392 × 10^−24^	GO:0002697
20	Positive regulation of lymphocyte mediated immunity	1.7601 × 10^−24^	GO:0002708

## Supplementary Materials

Supplementary materials can be found at http://www.mdpi.com/1422-0067/19/11/3311/s1.

## Abbreviations

SC	Serous carcinoma
GO	Gene ontology
EOC	Epithelial ovarian cancer
FIGO	Federation of gynecologists and obstetrics
ERBB	Erb-B2 Receptor Tyrosine Kinase
PI3K	Phosphoinositide 3-kinase
EAOC	endometriosis-associated ovarian carcinoma
GEO	Gene expression omnibus
MSigDB	Molecular signatures database
GSR	Gene set regularity
SD	Standard deviation
SVM	Support vector machine
AUC	Area under curve
CD74	CD74 Molecule
NK	Natural killer
WNT5A	Wnt family member 5A
NR1H3	Nuclear receptor subfamily 1 group H member 3
STAP1	Signal transducing adaptor family member 1
RORA	RAR related orphan receptor A
ZC3H12A	Zinc finger CCCH-type containing 12A
PLA2G10	Phospholipase A2 group X
IL33	Interleukin 33
CDKN2A	Cyclin dependent kinase inhibitor 2A
SYK	Spleen associated tyrosine kinase
MYB	MYB proto-oncogene, transcription factor
FOXJ1	Forkhead box J1
ZEB1	Zinc finger E-box binding homeobox 1
CD74	CD74 molecule
LGALS9	Galectin 9
ADAM8	ADAM metallopeptidase domain 8
GLI2	GLI family zinc finger 2
CD86	CD86 molecule
PRKCD	Protein kinase C delta
CORO1A	Coronin 1A
PTPN6	Protein tyrosine phosphatase, non-receptor type 6
MSH2	MutS homolog 2
EXO1	Exonuclease 1
SLC11A1	Solute carrier family 11 member 1
CD27	CD27 molecule
GATA3	GATA binding protein 3
GZMB	Granzyme B
IL2	Interleukin 2
TAA	Tumor-associated antigens
MHC	histocompatibility complex
TIL	tumor infiltrating lymphocytes
CD3	cluster of differentiation 3
CD8	cluster of differentiation 8
IL4	interleukin 4
IL13	interleukin 13
IL10	interleukin 10
M-CSF	macrophage colony-stimulating factor
GM-CSF	Granulocyte-macrophage colony-stimulating factor
IFN-γ	Interferon γ
TLR	Toll-like receptor
IL8	Interleukin 8
Th1	T helper 1
Th2	T helper 2
IL12	interleukin 12
TNF-α	Tumor Necrosis Factor
Treg	regulatory T cell
DIRAC	Differential rank conservation
TCGA	The cancer genome atlas
